# Influence of Gradual Damage on the Structural Dynamic Behaviour of Composite Rotors: Experimental Investigations

**DOI:** 10.3390/ma11122421

**Published:** 2018-11-29

**Authors:** Angelos Filippatos, Maik Gude

**Affiliations:** Institute of Lightweight Engineering and Polymer Technology (ILK), Technische Universität Dresden, 01307 Dresden, Germany; maik.gude@tu-dresden.de

**Keywords:** gradual damage behaviour, damage propagation, modal properties, composite rotor, structural dynamic behaviour

## Abstract

Fibre-reinforced composite structures subjected to complex loads exhibit gradual damage behaviour with the degradation of the effective mechanical properties and changes in their structural dynamic behaviour. Damage manifests itself as a spatial increase in inter-fibre failure and delamination growth, resulting in local changes in stiffness. These changes affect not only the residual strength but, more importantly, the structural dynamic behaviour. In the case of composite rotors, this can lead to catastrophic failure if an eigenfrequency coincides with the rotational speed. The description and analysis of the gradual damage behaviour of composite rotors, therefore, provide the fundamentals for a better understanding of unpredicted structural phenomena. The gradual damage behaviour of the example composite rotors and the resulting damage-dependent dynamic behaviour were experimentally investigated under propagating damage caused by a combination of out-of-plane and in-plane loads. A novel observation is the finding that a monotonic increase in damage results in a non-monotonic frequency shift of a significant number of eigenfrequencies.

## 1. Introduction

Composite materials provide high strength and stiffness-to-weight ratios and adjustable directional material properties. Due to such outstanding properties, there is a noticeable increasing interest in the use of composite materials for weight-relevant applications of complex-loaded structures. In particular, the constantly growing requirements for efficiency and reliability in modern high-performance rotors for gas, steam, and wind turbines, as well as ventilators, demand the increased application of advanced fibre-reinforced composites, as conventional materials are reaching their physical limits. Besides outstanding specific strength and stiffness properties, fibre-reinforced composites offer the significant advantage of cost-efficient manufacturing due to the feasibility of producing very complex, near-net-shaped fibre reinforcements for rotor components. In addition, their adjustable gradual damage behaviour is advantageous—in contrast to the classical metallic materials—as it allows for the development of rotors which are characterised by a gradual damage behaviour.

Due to this gradual damage behaviour, the remaining structural strength of composite rotors is generally not reduced critically, and a structural failure will often be prevented as long as the loads do not further increase. Notably, the gradual damage strongly affects the structural vibration behaviour, which means that an altered dynamic behaviour of the composite structure can be treated as a symptom of a new damage state, which can be of major relevance to advanced damage identification methods, especially for vibration-based diagnostic approaches.

### 1.1. Motivation

An unknown or incorrect understanding of the damage-dependent structural dynamic behaviour of composite rotors can lead to severe misinterpretations and error-prone decision making. Especially in damage identification methods, such as vibration-based diagnostics, a misclassification of the vibration response to a damage state can result in a catastrophic failure.

The goal of the current investigations is to gain information and knowledge regarding the effect of complex damage phenomena on the structural dynamic behaviour of composite rotors. As a result, the relation between the gradual damage behaviour of composite rotors and the resulting damage-dependent structural dynamic behaviour is described and depicted as frequency shifts of selected eigenfrequencies to the increasing damage.

### 1.2. State-of-the-Art

A thorough body of literature exists on the design and development of composite rotors, but it only partially addresses the complex gradual damage behaviour of composite materials. Furthermore, few investigations are found regarding the effect of the gradual damage behaviour on the structural dynamic behaviour of composite rotors. Even fewer are publications that investigate complex, non-monotonic changes in structural dynamic behaviour, such as modal properties, due to increased damage.

#### 1.2.1. Gradual Damage Behaviour of Composites

The increasing application of composite materials has led to numerous investigations on the gradual damage behaviour of composites, e.g., [1,2,3,4]. These investigations reveal that the gradual damage behaviour of composites is governed by a mixture of various fracture modes, e.g., inter-fibre failure, fibre failure, as well as delamination. The sequence of these modes depends notably on different fibre architectures and loading conditions [5,6]. Based on these investigations, novel material models combining multiple failure criteria and continuum damage mechanics have been developed to describe the non-linear stress–strain behaviour of composite materials due to damage propagation under different loading conditions [7,8,9,10,11,12]. These models characterise the interactions between different failure modes, as well as the resulting non-linear deformation process, and they have already been applied to the study of diverse fibre- and textile-reinforced plastics [13,14]. Particularly, they have been appropriately validated in experimental studies for specimens with a simple geometry and homogeneous loading conditions [5,15].

The gradual damage behaviour of composite materials, occurring from physical-based phenomena, results in local changes in stiffness and damping. This leads to a noticeable sequential alteration of the dynamic behaviour: specifically, to a shift of the eigenfrequencies and of the modal damping ratio.

#### 1.2.2. Composite Rotors

**Figure 1:**
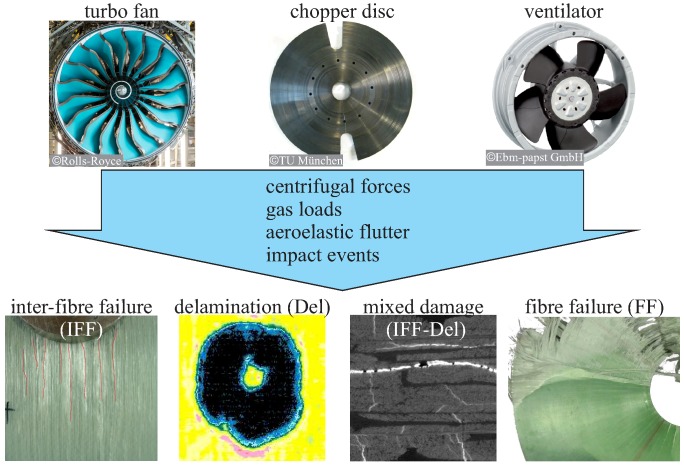
Examples of composites in high-performance rotors, turbofan aero engines, wind turbines, and ventilators, and typical failure phenomena.

For the description of the gradual damage behaviour of composite rotors caused by operational loads or unpredicted loads, such as impacts, verified damage mechanics models for composite materials are already available [5,16]. Typical failure modes for composite rotors are mainly inter-fibre failure from in-plane loads [17] and a mixture of inter-fibre failure, delaminations, and fibre failure from unexpected impact loads [18,19], as depicted in Figure 1. However, the application of these damage mechanics models to rotor-typical loading conditions with consideration of the gradual damage behaviour and the resulting structural dynamic behaviour has not been thoroughly investigated.

Especially in the aerospace industry, increasing economical demands combined with high performance requirements have led to substantial breakthroughs in the design and development of high-speed composite rotors, such as rotor blades [20,21,22,23]. The main focus has been directed toward the in-plane and out-of-plane dynamic behaviour, as well as the anisotropic material damping of undamaged composite rotors [24,25,26,27,28]. However, the gradual damage behaviour of composite rotors under unexpected loads has not been the main scope of the investigations of many researchers [29,30], and even less examined is the relation between gradual damage behaviour and the corresponding dynamic behaviour of composite rotors.

#### 1.2.3. Non-Monotonic Change in Eigenfrequencies

A number of authors have reported a non-monotonic change in eigenfrequencies in composite structures. In particular, the effect of low-impact-related delaminations has been investigated [31,32,33,34], mainly due to local thickening accompanied by matrix cracking. Other similar investigations, however, did not identify this effect and reported a continuous reduction in the first eight eigenfrequencies with increasing delamination [35,36].

### 1.3. Aim and Outline of the Paper

The aim of this paper is to investigate the structural dynamic behaviour of composite rotors subjected to propagating damage from a combination of out-of-plane and in-plane loads.

A thorough experimental study was performed in order to investigate the gradual damage behaviour of composite rotors and the relation between damage and vibration behaviour. An elementary rotor geometry was selected with a representative fibre architecture for a Cartesian-orthotropic material behaviour. Consequently, a test matrix was defined, including representative damage sequences and practice-relevant load conditions. Then, the rotors were investigated with their initial damage as well as propagating damage, where both the resulting damage and the dynamic behaviour were estimated. The resulting damage was subsequently evaluated using a variety of non-destructive testing methods in order to assess the type and extent of the inflicted damage.

For determining the dynamic behaviour of the composite rotors, experimental modal analysis tests were conducted, for which an impact excitation was achieved using an electrodynamic shaker with a mounted steel impactor. Finally, the damage-dependent dynamic behaviour of selected rotors was experimentally estimated, and the results are presented and evaluated.

## 2. Composite Disc Rotors under Rotor-Typical Load Conditions

**Figure 2:**
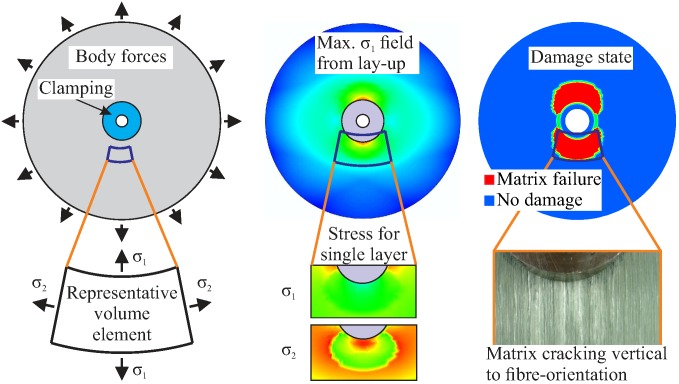
Rotor subjected to complex loading (**left**); data taken from a finite element model (FEM) of the calculated stress field (**centre**) and in-plane anisotropic damage state (**right**).

High-speed composite rotors undergo complex, inhomogeneous, and variable stress conditions induced by centrifugal forces, which are mainly characterised by multi-axial tension and shear loads, as well as by further operational loads. The complex fibre-matrix architecture and its corresponding progressive damage behaviour increase the structural complexity, as shown in Figure 2. Furthermore, unpredicted impact loads can cause unexpected out-of-plane compression loads that lead to inter-fibre failure and delaminations.

### 2.1. Load-Adapted Fibre Architectures

High-speed composite rotors using multi-axial non-crimp fabrics (NCF) as reinforcement are nowadays the state of the art in the industry. A particular advantage of NCF in the field of textile semi-finished products is the absence of yarn crimping and a high variability. These advantages, in combination with a multilayer-manufacturing capability, make this fabric type suitable for the efficient production of high-performance rotors. Another important feature of NCFs is an adjustable damage behaviour that—in contrast to classical metallic materials—allows for a design of composite rotors with gradually progressing damage and predictable alterations of the resulting anisotropic material stiffness [29,37]. In particular, NCFs offer high specific stiffness and strength, as well as an adjustable energy absorption capacity. Currently, NCFs are suited for various applications, with reported examples that include composite chopper discs, saw discs, and flywheels [38,39,40,41]. The investigation of rotors with a multi-axial fabric of endless glass fibres could, therefore, benefit the aforementioned industries.

### 2.2. Stress Distribution

**Figure 3:**
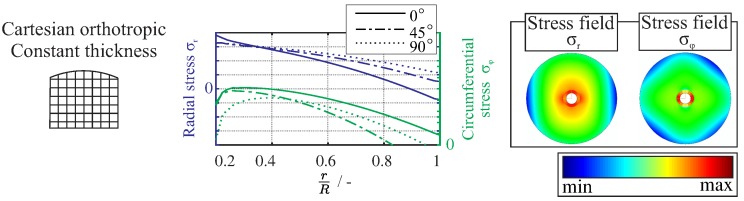
Illustration of rotors with different fibre architectures subjected to centrifugal forces; data taken from a finite element model with the calculated radial stress distribution (**centre**) and in-plane stress fields (**right**).

Problem-adapted semi-analytical calculation models have already been developed for the basic analysis of the deformation and failure behaviour of composite rotors [42]. These models provide an important contribution using elementary geometries, such as disc rotors, to the development and optimisation of high-performance composite rotors. The structural analysis of Cartesian-orthotropic disc rotors can be achieved by means of closed mathematical solutions and for complex lay-ups using finite element methods, as shown in Figure 3.

For the mathematical description of laminated rotors in the Cartesian coordinates x,y, the methods of conformal mapping and complex stress functions are applied [43]. The resulting stress and displacement fields are dependent on the fibre orientation ϕ, which can be seen in the following relations,

(1) $$\sigma_{i},u_{j}∼\rho \cdot \omega^{2}\cdot \left(x^{2}+y^{2}\right)$$

(2) $$\sigma_{i},u_{j}=F\left(\phi ,\omega ,\ldots \right)$$

where $\sigma_{i}=\sigma_{x},\sigma_{y},\tau_{xy}$ are the stress components; ρ is the density; $u_{j}=u_{x},v_{y}$ are the displacement components; and ω is the angular velocity.

In the case of the Cartesian-orthotropic composite disc rotors, the structural behaviour can be determined on the basis of plane stress conditions using the method of angle-preserving mapping and complex-valued stress functions. However, a rotationally symmetric load will result in a non-rotationally symmetrical stress state. Therefore, under complex in-plane loading from a centrifugal load $\sigma_{r}^{+}$, tensile stresses are mainly combined with intralaminar shear stresses ($\tau_{12}$). This results in non-rotationally symmetrical damage evolution, which is mainly inter-fibre failure under different ply-dependent angles.

## 3. Material Selection of Fibre Architecture and Manufacturing of Rotors

For the experimental investigation of the damage behaviour of composite rotors and their resultant dynamic behaviour, a multi-ply multi-axial fabric was selected, resulting in an in-plane Cartesian-orthotropic behaviour.

**Figure 4:**
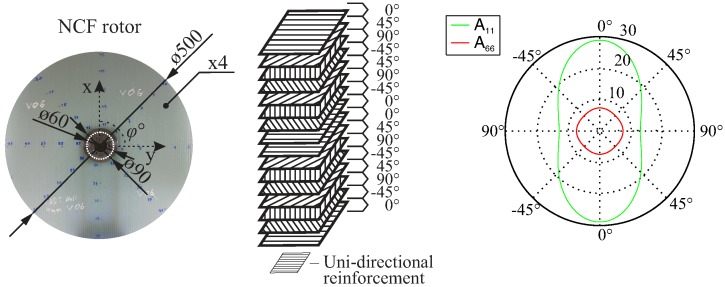
Geometry of a non-crimp fabric (NCF) rotor (**left**) with the lay-up (**centre**) and the corresponding homogenised directional stiffness properties A_11_, A_66_, resulting in an in-plane Cartesian-orthotropic behaviour (**right**).

The selected NCF-fibre architecture is composed of a glass-fibre, non-crimp, multi-ply, and multi-axial fabric [43]. The fabric reinforcement has an area density of 1.90 kg/m^2^ and a ply thickness of 1 mm. Each of the $\left(0^{\circ },-45^{\circ },90^{\circ },+45^{\circ }\right)$ layers have a thickness of (0.48,0.23,0.05,0.23) mm, respectively. The composite lay-up consists of four such fabrics, $(\left(0^{\circ }/-45^{\circ }/90^{\circ }/+45^{\circ }\right)/(-45^{\circ }/90^{\circ }/45^{\circ }/0^{\circ })/\left(0^{\circ }/-45^{\circ }/90^{\circ }/45^{\circ }\right)/\left(-45^{\circ }/90^{\circ }/45^{\circ }/0^{\circ }\right))$, as shown in Figure 4, resulting in a total laminate thickness of 4 mm. The inner and outer diameters of the rotor are 60 mm and 500 mm, respectively. The lay-up results in an in-plane orthotropic behaviour, and it was selected in order to achieve polar non-symmetrical damage evolution caused by applied rotational loads. This material was extensively investigated in previous research projects, where the results indicated a gradual damage behaviour [44].

A total of five NCF rotors were manufactured, and their dynamic behaviour was experimentally investigated. The fabrication of the composite rotors was carried out using a state-of-the-art vacuum-assisted resin transfer moulding process, with the epoxy resin MGS-RIM-135 and process parameters similar to those by previously performed investigations [44].

## 4. Method for the Investigation of Representative Damage Sequences

**Figure 5:**
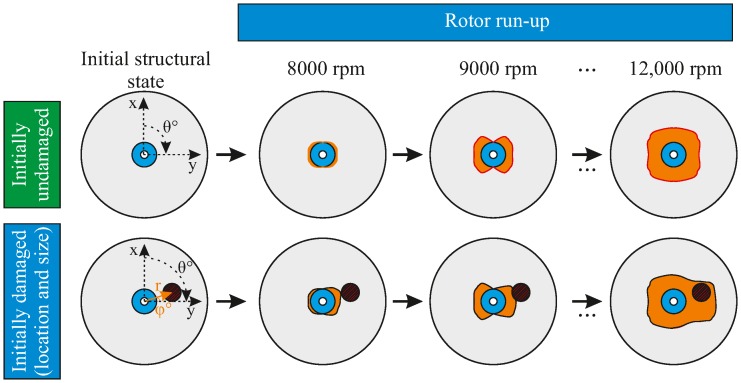
Illustration of two different damage sequences resulting from damage propagation during multiple rotor run-ups: one sequence without any initial damage (**top**), and one having initial damage due to an out-of-plane load.

The gradual damage evolution in a composite rotor throughout its entire designed operational lifetime includes both predicted and unpredicted load events, which can result in a structural failure before the end of its designed lifetime. A qualitative example of damage accumulation in a rotor under the influence of unpredicted events was reported in [45]. The current approach utilised and further developed this work by specifying different load events. First, an out-of-plane load was introduced, resembling an impact event, which resulted in the initial damage at different locations using different load levels, as shown principally in Figure 5. Then, the composite rotor was run at different rotational velocities in order to induce further damage by increasing in-plane centrifugal loading.

Using such a combination of out-of-plane and in-plane loads, representative damage sequences can be generated with multiple structural states, for which their damage state and their dynamic behaviour can be investigated. At first, a sequence without initial damage was investigated for three nominally equal NCF rotors. Furthermore, two different sequences with the same initial damage at different positions were tested, each for a single NCF rotor. The boundary conditions always have a significant impact in the resulting vibration behaviour. In order to avoid such an effect, each rotor was clamped at the beginning of the experiments, and all the screws were locked and sealed to prevent loosening from the vibration. After a complete investigation of the damage sequence for each rotor, the clamping was removed and the next rotor was investigated. Each investigated rotor was run up at different rotational velocities, as shown in Table 1.

### 4.1. Determination of Damage-Initiating Loads

**Figure 6:**
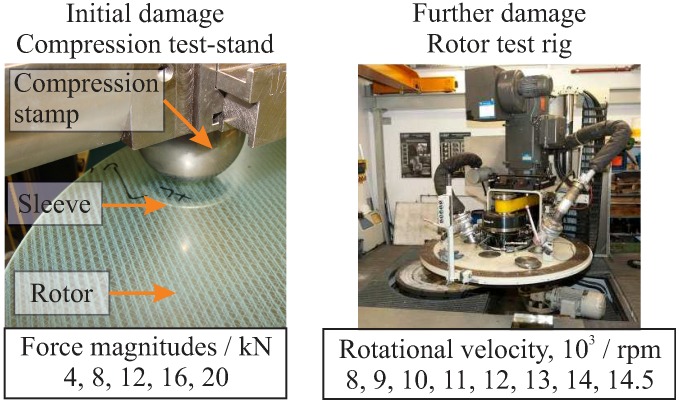
The compression test stand (**left**) and the rotor test rig (**right**) used for sequential damage introduction.

In order to create rotor-typical damage from out-of-plane and in-plane loading, two damage-initiating loads were selected. Specifically, an out-of-plane compression load and an in-plane centrifugal load were introduced (Figure 6).

#### 4.1.1. Out-Of-Plane Compression Loading for Damage Initiation

In order to introduce an impact-like damage to the rotors, a typical testing machine, ZWICK, equipped with a ball stamp and a sleeve base was used, as shown in Figure 6. The force-controlled testing machine presses the ball stamp and the rotor surface in order to introduce barely visible damages to different regions of the investigated rotors. The ball stamp has a diameter of 80 mm, with a sleeve inner diameter of 63 mm and outer diameter of 75 mm. Five independent compression loads were applied with different load magnitudes between 4 kN and 20 kN, as shown in Table 2.

#### 4.1.2. In-Plane Loading from Multiple Rotor Run-Ups for Damage Propagation

In-plane loading was experimentally introduced by sequential run-ups of the rotor, using the available high-speed rotor test rig BI4U at the Institute of Lightweight Engineering and Polymer Technology (ILK), shown in Figure 6. In order to avoid any changes in the temperature-dependent material properties of the matrix due to the friction between the rotor and the air, a technical vacuum was applied in the range of 2.4–2.7 mbar.

The damage initiation of NCF rotors was approximately determined at 8000 rpm in the progressive run-up tests, in which the rotors were run up in steps of 1000 rpm. The rotors were first accelerated to 8000 rpm with an angular acceleration of 3 rad/s^2^, then rotated at the defined speed for a time period of 120 s and subsequently decelerated at 3 rad/s^2^ to a stand-still. With every run-up, the body forces increase, causing the propagation of the in-plane damage of the rotors.

The used rotor test rig was mounted with a fail-safe monitoring system, causing an automatic shutdown of the test rig when a shaft vibration of 350 μm is reached. Due to the propagating damage, different imbalances accumulated for the two rotor types. These imbalances are reflected by increasing vibration values in the shaft. Therefore, the NCF rotors were sequentially run-up in steps of 1000 rpm until the rotor test rig was automatically shut down due to a shaft vibration of 350 μm.

For the NCF rotor I.1, without any initial damage, the test was completed at 14,500 rpm, similar to the NCF rotors I.2 and I.3 which completed testing at 14,000 rpm (see Table 1). However, for the NCF rotors II and III, which had initial damage, the rotor test rig shut down at 12,000 rpm and 12,600 rpm, respectively.

### 4.2. Evaluation of the Inflicted Damage

A variety of non-destructive evaluation methods were applied in order to perform a phenomenological assessment of the type and extent of the inflicted damage. Characterisation of the initial damage and the propagating damage was performed, and the predominant failure modes were identified for every case. Three non-destructive evaluation methods were applied, with each method identifying different aspects of the failure modes with a measurement resolution at the μm scale:

- High-resolution visual inspection for the identification of spatial inter-fibre cracks due to the in-plane load;
- Ultrasonic testing for the evaluation of the form and size of delaminations;
- Computer tomography for the identification of single inter-fibre failures, resin-rich areas, and the number of delaminations through the thickness.

#### 4.2.1. High-Resolution Optical Inspection

As the fibre architecture has a refraction index similar to that of the epoxy matrix, which amounts to 1.548–1.552, it is possible to use optical inspection to identify the inflicted damage [25]. A high-resolution optical camera Nikon D3100 was therefore used with an optical resolution of 4608×3072 pixels. After each applied loading, the state of the rotor was photographically documented in order to capture the inflicted damage.

**Figure 7:**
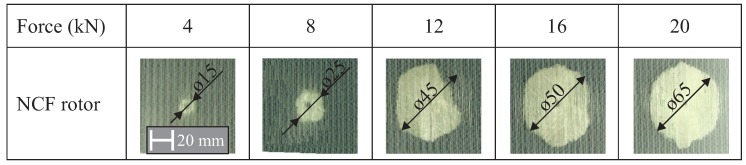
Optical resolutions of five different delaminated areas for an NCF rotor of five different load magnitudes.

The transparency of the investigated composite rotors allows for the monitoring of both the crack densities and the existence of delaminations from the initial impact using the optical method with a high-resolution camera, as is shown in Figure 7. At low magnitudes of up to 4 kN, a small damage is observed, and an increase in the delaminated area is observed between 8 kN and 16 kN. For a magnitude of 20 kN, the applied delamination has the same area as in the case of 16 kN.

**Figure 8:**
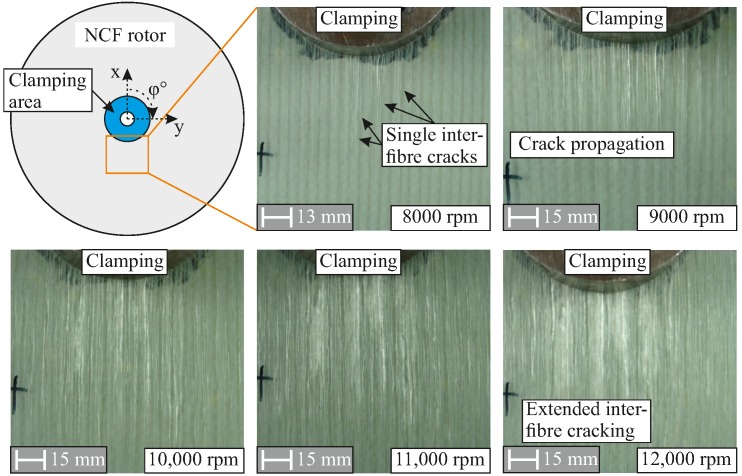
Typical damage due to a run-up of an NCF rotor after different rotational velocities, resulting in an increasing number of spatial matrix damages.

By using the photographically documented damaged states of different areas of an NCF rotor, inter-fibre cracks are identified in the respective main fibre orientations, as shown in Figure 8. With increasing damage, inter-fibre cracks further develop in each layer, becoming visible as crack lines in their respective directions. All rotors exhibit the same damage initiation from the to run-up—specifically, inter-fibre damage—at similar rotational velocities due to the used matrix system. The selected fibre architecture of the NCF rotors and the corresponding Cartesian-orthotropic material behaviour result in a non-rotational symmetrical damage propagation, as shown in Figure 8.

#### 4.2.2. Ultrasonic Testing for the Evaluation of Delaminations

**Figure 9:**
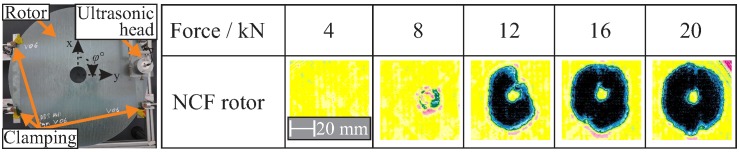
Overview of the ultrasonic results after out-of-plane compression loads with different force magnitudes for NCF rotors, where the formation of delaminations is observed by 8 kN.

The ultrasonic test setup is shown in Figure 9 (left), where an NCF rotor is clamped at four points and an air-coupled ultrasonic test takes place in a modular ultrasonic inspection system from the company Dr. Hillger, type AirTech-4000, with a maximum resolution of 125 μm.

Due to the increased inherent material damping of composites, the testing range was approximately 250 kHz. As a result, the spatial resolution is decreased, making ultrasonic testing more suitable for the delamination investigation than for single, isolated inter-fibre failures [46]. Therefore, inter-fibre cracks and fibre fracture paths are difficult to detect, because their reflecting surface is not wide enough, in contrast to delaminations [47].

The relationship between the investigated compression loads and the resulting damaged area is shown in Figure 9. Clearly distinguishable damage regions are highlighted by the black and blue colours in the ultrasonic images. Based on these results, the delamination shape is quantified as a circular area. The position of the delamination within the thickness and the existence of multiple delaminations were determined using the subsequently presented computer tomography scans.

#### 4.2.3. Computer Tomography Investigations

Computer tomography (CT) is predestined for the material assessment of composites because it is a non-destructive approach and provides a measurement resolution at the µm scale. Therefore, CT scans were taken for the identification of single inter-fibre failures, resin-rich areas, and the number of delaminations. In this work, conventional investigations were performed using the CT machine Nanotom 180 nF from the company GE Sensing & Inspection Technologies.

**Figure 10:**
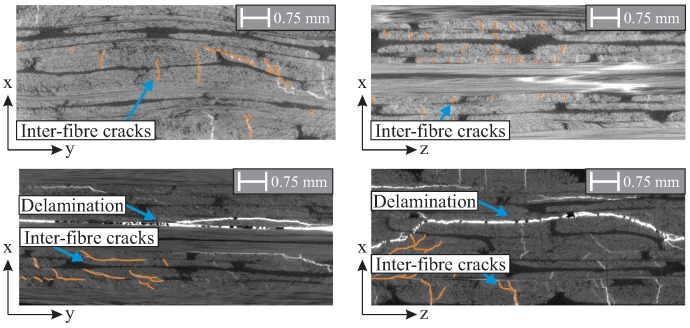
Computer tomography picture of an NCF rotor after an in-plane load and the corresponding damage, where inter-fibre cracks are identified as the predominant failure mode (**top**); and with the initial damage caused by a compression load of 16 kN, resulting mainly in delaminations and inter-fibre cracks (**bottom**).

The applied in-plane loads result in the formation of inter-fibre cracks, as shown in Figure 10. For the damage to the NCF rotor III caused by an applied rotational velocity of 12,000 rpm, an extended inter-fibre cracking in multiple layers is observed transversely to the main fibre orientation, as shown in Figure 10 (top). The inter-fibre cracks are distributed among many layers of the lay-up.

For the NCF rotor III damage caused by an out-of-plane compression load of 16 kN, the characteristic failure types are identified as delaminations and inter-fibre cracks, as shown in Figure 10 (bottom). A main delamination is formed for the NCF rotors near the middle position of the lay-up. Furthermore, multiple smaller delaminations and inter-fibre cracks are evident between the layers.

#### 4.2.4. Identification of Predominant Failure Modes

A mixture of different failure modes are identified for both loading conditions. An overview of the identified resulting damage is shown in Table 3. Notably, the propagating damage caused by rotor run-up starts approximately at 40% of the maximum applied in-plane load and comprises mainly inter-fibre cracks. Fibre failure is not detected until the total failure of the rotors, and the gradual damage behaviour is confirmed for all rotors.

## 5. Results and Discussion of the Investigated Dynamic Behaviour of Composite Rotors

The damage-dependent dynamic behaviour of the investigated rotors was experimentally estimated, and selected results are presented. For each investigated damage state, an experimental modal analysis was performed using a self-developed excitation system combined with a contactless laser-scanning vibrometer (LSV) (Polytec, Type PSV-400).

### 5.1. Experimental Modal Analysis

**Figure 11:**
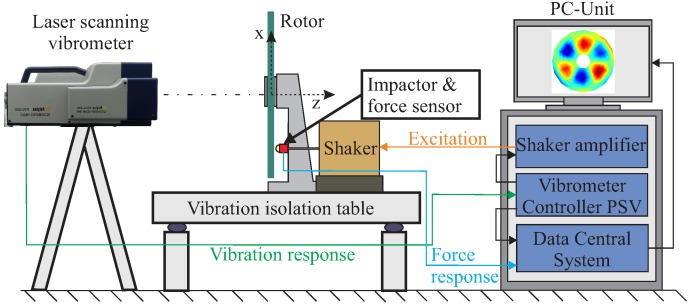
Experimental setup for the modal analysis of clamped composite rotors; the impact excitation was achieved using an electrodynamic shaker with a mounted steel impactor; the vibration response was measured using a laser-scanning vibrometer (Polytec, Type PSV-400).

For the excitation for modal analysis, an optimised burst signal was sent to an electrodynamic shaker, where a steel impactor and a force sensor were mounted, as shown in Figure 11, causing a stroke of the impactor. In this way, double hits are eliminated and excitation uncertainties are reduced, which is the main advantage of impact hammer modal analysis. A further advantage of the developed excitation strategy is the reproducibility. Between sequential measurements by the force sensor, a deviation of 0.1% was calculated, showing a precise excitation capability. The excitation point was carefully chosen in order to avoid exciting the disc in a certain mode shape. To achieve this, the rotor was excited in previous investigations at several points in order to examine the vibration responses in the frequency domain. The chosen excitation point showed good results, and the mode shapes could be experimentally determined. Furthermore, a finite element model (FEM) was developed and used for for cross-checking the eigenmodes. Using the results from the FEM, it could be verified that all numerically identified mode shapes in the frequency range of interest were also experimentally measured, and no eigenmodes were omitted.

For acquiring the vibration response and the resulting mode shapes of each eigenfrequency, the roving measurement point method was selected for the measurement of the resultant vibration response due to the existing equipment: the developed combined excitation system and the LSV [48].

**Figure 12:**
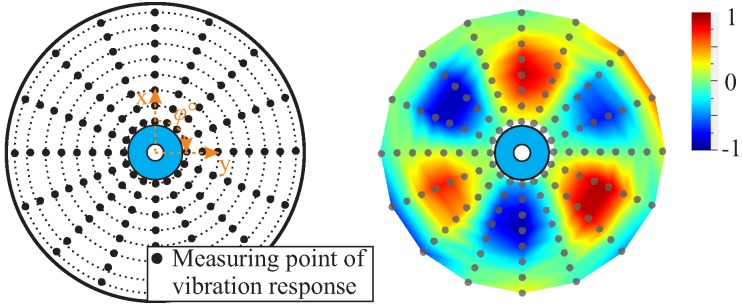
Measuring mesh with a pattern of 128 measuring points for the determination of the vibration response of each rotor (**left**) and an experimentally determined mode shape (3,1,0), with a colour plot of the normalised measured surface speed (**right**).

For the experimental estimation of the vibration response of each rotor, a pattern of 128 measuring points was defined, as shown in Figure 12. This number of measuring points serves to experimentally determine local mode shapes at higher frequencies.

Based on the position of the measurement points, different components of the vibration response were measured using the LSV, providing information regarding the excited eigenfrequencies at the measured points, with selected settings shown in Table 4. As measured signals were superimposed with noise, subsequent measurements were taken, and magnitude averaging was implemented to reduce the noise-to-signal ratio. For each determined response of *N* samples, the magnitudes of their complex values were calculated, added, and subsequently divided by *N*, as shown in Equation (3) [48]:

(3) $${\bar{P}}_{yy}=\frac{1}{N}\;\cdot \;\sum_{i=1}^{N}\left|P_{yy}\left(i\right)\right|\;.$$

### 5.2. Assessment of the Damage-Dependent Dynamic Behaviour

The relation between the applied in-plane loads and the corresponding shift of eigenfrequencies due to the damage increase was investigated for five different damage sequences. For each damage sequence, a total of 16 eigenfrequencies were investigated for five NCF rotors. For each type of rotor, a normalised in-plane load $F_{c}^{\prime }$ was introduced,

(4) $$F_{c}^{\prime }={\left(\frac{n_{rpm}}{n_{rpm}^{max}}\right)}^{2}$$

where each rotational velocity $n_{rpm}$ is divided by the maximum rotational velocity achieved $n_{rpm}^{max}$ for each sequence, as was shown previously in Table 1.

The frequency shift for each eigenfrequency $EF_{i}$ was determined for each damage state $S_{j}$ under a rotational in-plane load as

(5) $$\Delta f\left(EF_{i}|S_{j}\right)=f\left(EF_{i}|S_{j}\right)-f\left(EF_{i}|S_{0}\right),$$

where $EF_{i}|S_{j}$ is the eigenfrequency at the $S_{j}$ state, and $EF_{i}|S_{0}$ is the eigenfrequency value at the $S_{0}$ state. For the two damage sequences where multiple rotors were investigated, i.e., NCF rotor I, a mean frequency shift was calculated and fitted using a third-degree polynomial.

**Figure 13:**
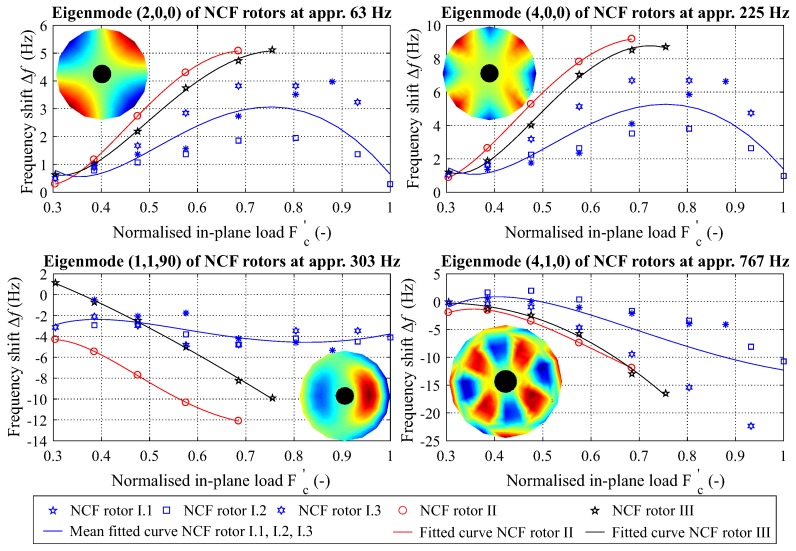
Shift of the eigenfrequencies of all investigated NCF rotors for two eigenmodes shown as an example: one with a non-monotonic change in the eigenfrequency (**left**) and one with a monotonic decrease in the eigenfrequency (**right**).

Two different types of frequency shifts for each EFF can be observed for all investigated damage sequences, as shown in Figure 13 for four typical eigenmodes. First, the eigenfrequencies of the mode shapes (1,1,90) and (4,1,0) remain constant and slightly increase, followed by a typical frequency decrease with the increase in damage, as shown in Figure 13 (right).

However, a different behaviour is exhibited for the eigenfrequencies of the mode shapes (2,0,0) and (4,0,0). First, the frequency increases, and with increasing damage, it reaches a maximum, as shown in Figure 13 (left). Then, with further damage increase, the eigenfrequency change decreases. The aforementioned behaviour between different types of eigenmodes is visible for both sequences without initial damage, NCF-rotor I; those with initial damage, NCF-rotor II and NCF-rotor III; and for other investigated eigenmodes.

**Figure A1:**
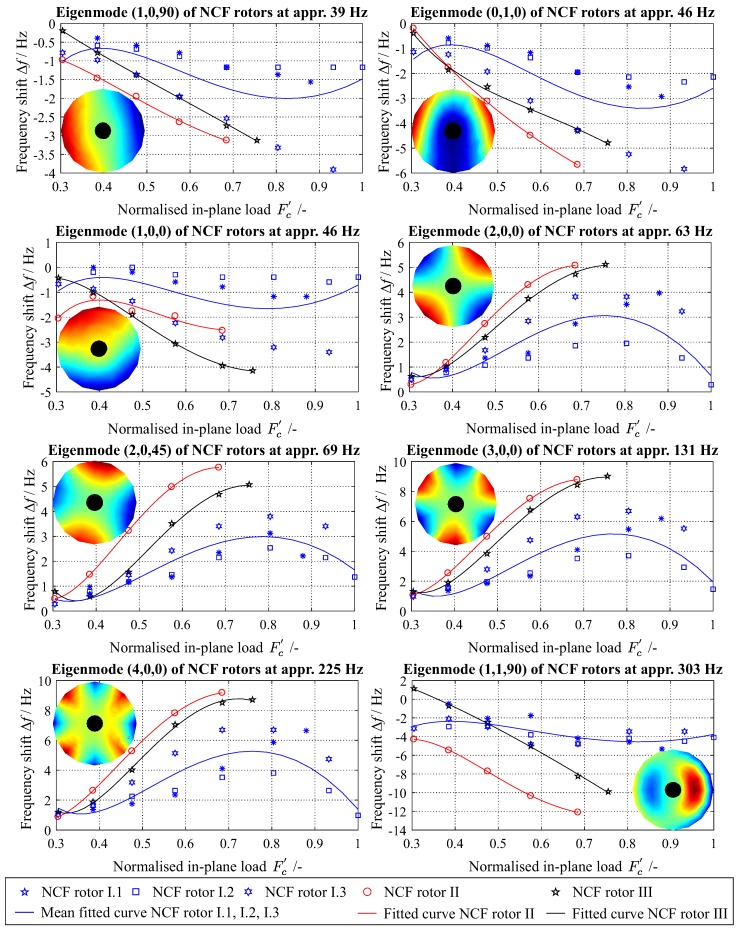
Experimentally determined frequency shift of investigated NCF rotors.

**Figure A2:**
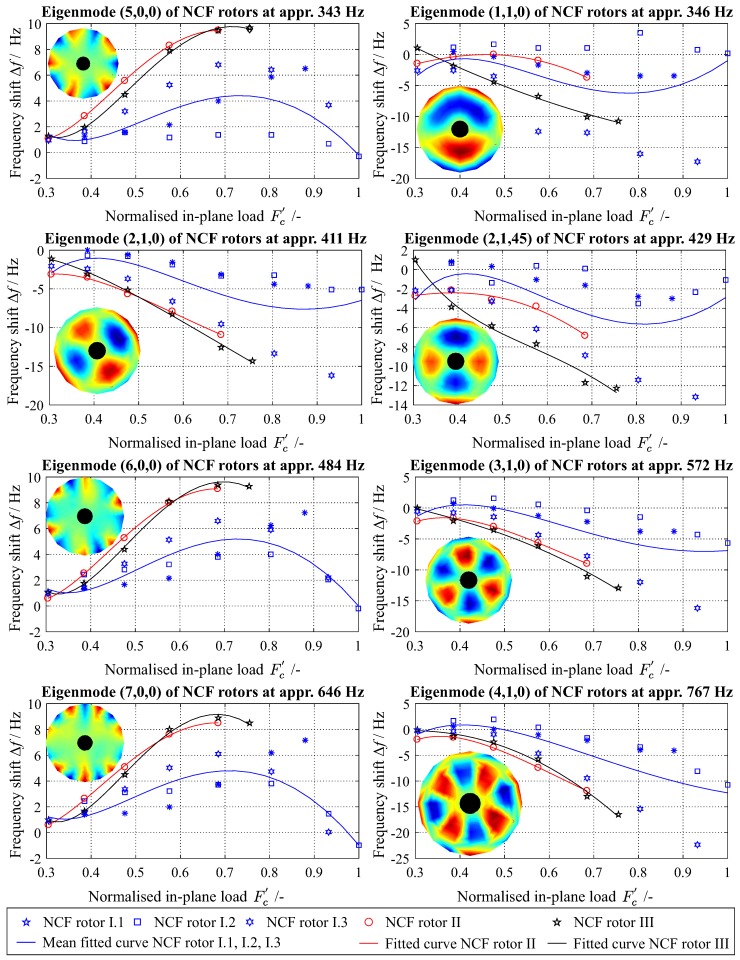
Experimentally determined frequency shift of investigated NCF rotors.

Based on these results, it can be deduced that a monotonic increase in damage can result in a non-monotonic frequency change for 10 out of 18 eigenfrequencies, a percentage of 55% of all investigated eigenmodes, as shown in Figure A1 and Figure A2.

This effect is observed for all investigated sequences, with and without initial damage. Numerical investigations show that the reasons for the non-monotonic behaviour could be coupling effects, a combination of pre-stress effects from the curing process, the type of damage propagation combined with the geometry, and the specific mode shapes. The scatter in the NCF-rotor I mainly comes from manufacturing deviations due to the relatively low stiffness of the upper part of the mould, which was used for the manufacturing of the rotors. A scatter between nominally same rotors will always be there, and further investigations are required to address whether this scatter is due to only manufacturing deviations or whether it also arises from the damage evolution sequence itself.

## 6. Conclusions

A thorough experimental investigation was conducted in order to analyse the gradual damage behaviour of composite rotors. Representative damage sequences were generated by applying both in-plane and out-of-plane loads, and they are considered to be sequences of distinct structural damage states. It was experimentally determined that the investigated composite rotors were damaged by the applied forces but that they are still operational even after a substantial degree of inter-fibre failure.

For identifying the experimental damage, a number of investigations were performed for each generated structural state. First, non-destructive testing was applied in order to identify the inflicted damage. Based on three different non-destructive evaluation methods, the damage was evaluated, and the predominant failure types were identified and described. Then, the structural dynamic behaviour was estimated using experimental modal analysis, and the modal properties were determined for each damage state. A novel observation is reported: it was found that a monotonic increase in damage can result in a non-monotonic frequency shift of a significant number of eigenfrequencies for all investigated rotors.

This work provides the basis for further, detailed investigation of composite structures to study the non-monotonic change in eigenfrequencies for different fibre architectures, as well as varying loading and boundary conditions. Further ongoing investigations show that for these kinds of composite disc rotors, similar effects are evident for different laminates. Some preliminary numerical investigations on carbon fibre-reinforced epoxy plates also show promising results, although, to date, they are not yet published as further in-depth study is still required.

## Figures and Tables

**Table 1:** Overview of the experimentally investigated sequences for all types of rotors, with and without initial damage and under different rotational velocities.

Name	Initial Damage	Applied Rotational Velocity $10^{3}$ rpm										
	F (kN), *r* (mm), *θ* (°)	0	8	9	10	11	12	12.6	13	13.6	14	14.5
Rotor I.1	-	x	-	x	x	x	x	-	x	-	x	x
Rotor I.2	-	x	-	x	x	x	x	-	x	x	-	-
Rotor I.3	-	x	x	x	x	x	x	-	x	-	x	-
Rotor II	16, 100, 90	x	x	x	x	x	x	-	-	-	-	-
Rotor III	16, 100, 0	x	x	x	x	x	x	x	-	-	-	-

**Table 2:** Overview of the applied parameters for the initial damage using a force-controlled compression test.

Load Parameters	Unit	Values
Introduced force	kN	4, 8, 12, 16, 20
Test speed	mm/min	2
Compression stamp diameter	mm	80
Sleeve inner diameter	mm	63
Sleeve outer diameter	mm	75

**Table 3:** Overview of the identified resulting damage for both loading types.

Loading Type	Resulting Damage
Initial out-of-plane compression	Inter-fibre cracks
	Delamination
	Isolated fibre cracks at 20 kN
Centrifugal in-plane loading	Inter-fibre cracks
	Spatial inter-fibre crack distribution

**Table 4:** Overview of the applied settings for the experimental modal analysis.

Setting Parameters	Selected Setting
Measuring points	128
Type of excitation	Impact
Sample frequency	6.4 kHz
FFT settings	Triple magnitude averaging, 2.5 kHz, 12,800 lines, rectangular window
Type of vibrometer	Polytec, Type PSV-400

## Abbreviations

The following abbreviations are used in this manuscript:

NCF	Non-crimp fabric
FEM	Finite element model
ILK	Institute of Lightweight Engineering and Polymer Technology
CT	Computer tomography
LSV	Laser-scanning vibrometer
EF	Eigenfrequency
